# A Rail Profile Measurement Method Based on Polarization Fusion Imaging

**DOI:** 10.3390/s25113489

**Published:** 2025-05-31

**Authors:** Qiang Han, Xinxin Zhao, Jing Shi, Shengchun Wang, Peng Dai, Ning Wang, Le Wang

**Affiliations:** Infrastructure Inspection Research Institute, China Academy of Railway Sciences Corporation Limited, Beijing 100081, China

**Keywords:** polarization fusion, underexposure, rail profile, image fusion

## Abstract

The smooth area on the rail surface causes abnormal exposure in the laser section image, resulting in measurement errors of the rail profile. To address this issue, a novel rail profile measurement technique based on polarization fusion imaging is proposed. A polarized camera is utilized to acquire the four-directional polarization component images, Stokes parameter images, linear polarization angle images, and linear polarization degree images of the rail laser section. A polarization image data fusion algorithm based on Segmented Random Sample Consensus (S-RANSAC) is designed using these images as data sources, and the optimal rail profile fitting curve is obtained. Experimental results demonstrate that the proposed method can still obtain accurate and effective rail profile data in regions where traditional methods fail to capture profile data. Compared with the traditional method, the measurement error of the rail profile is reduced from 0.137 mm to 0.081 mm, and the measurement accuracy is improved by 40.9%. Evidently, this method avoids data loss in key areas of the rail profile caused by local underexposure, thus significantly enhancing the measurement accuracy. This method can provide a valuable reference for high-precision measurement of the rail profile under complex working conditions.

## 1. Introduction

It is widely acknowledged that changes in the rail profile directly impact the safe operation of railways. As a vital means of railway operation and maintenance, rail profile detection is instrumental in understanding the service condition of the rail. Based on this understanding, more effective rail grinding can be carried out [1,2,3]. Rail profile detection refers to the process of comparing the measured rail profile data with the standard rail profile data. This comparison helps to obtain parameters such as vertical wear and side wear of the rail [4]. Currently, rail profile detection mainly falls into two categories: contact-type detection and non-contact-type detection. Specifically, contact-type detection has disadvantages such as low detection efficiency and high labor costs. This is because it requires probes to be in contact with the rail. In contrast, non-contact-type detection aims to extract rail profile data from the intensity information of the reflected light on the rail surface. The line-structured light profile detection technology is a typical non-contact-type detection technology. The line-structured light rail profile detection technology, based on the triangulation measurement principle enables real-time acquisition of rail profile information. Due to its high speed, high precision, and non-contact nature, it is extensively used for dynamic rail profile detection at home and abroad [5,6,7].

However, influenced by harsh working conditions, the rail will undergo some changes after a period of operation. These changes include increased surface roughness, the presence of foreign matter on the surface, and rust on the rail head and the light stripe area of the rail. These surface changes will affect the energy distribution of the reflected light on the rail surface, leading to abnormal energy distribution. For example, the light stripe area of the rail head has a relatively smooth surface, strong specular reflection ability, and very weak diffuse reflection ability. Most of the incident light energy is distributed near the specular reflection direction. Only a small amount of diffuse reflection light is collected by the camera. The traditional line-structured light rail profile detection technology employs the diffuse reflection light as the measurement signal. The specular reflection light is considered the interference signal to obtain the intensity information of the reflected light on the rail surface. For the smooth light stripe area of the rail, when the traditional light rail profile detection method is used to acquire the light intensity image of the rail profile, the phenomenon of underexposure occurs.

Accurately extracting the center of the light stripe is crucial for ensuring the accuracy of rail profile detection [8]. Many scholars have conducted extensive research on the innovation and improvement of light stripe center extraction algorithms [9,10,11]. In summary, light stripe center extraction algorithms are mainly divided into geometric center extraction algorithms and energy center extraction algorithms [12]. Geometric center extraction algorithms are based on edge information, threshold information, or refinement techniques. They are applicable to simple working environments and situations where the requirements for measurement accuracy are not high. As prevailing light stripe center extraction algorithms, energy center extraction algorithms are based on the center-of-gravity, directional template, or maximum point. They are suitable for harsh working conditions, objects with complex shapes, and scenarios where high-precision measurement is required. The underexposed areas of rail profile light stripe images are characterized by weak light stripe energy, low contrast, and low confidence in the light stripe center. In such underexposed areas, even with more accurate light stripe center extraction algorithms like the center-of-gravity algorithm and the Steger algorithm, accurate rail profile information cannot be obtained. Moreover, when the light stripe energy is extremely low, the light stripe cannot be detected. As a result, it gives rise to partial loss of profile data and reduces the accuracy of profile detection [13,14,15,16]. Although the underexposure problem of the light stripe area can be mitigated to some extent by prolonging the exposure time, it will cause overexposure of light stripes in the normal area of the same image. This, in turn, affects the overall profile detection accuracy.

Traditional imaging technology can only acquire light intensity information. In contrast, the information obtained by polarization imaging detection technology is expanded from three dimensions (light intensity, spectrum, and space) to seven dimensions (light intensity, spectrum, space, polarization degree, polarization angle, polarization ellipticity, and rotation direction). The additional polarization information is often used to improve the imaging quality of the measured object. Many scholars have conducted extensive research on polarization imaging technology. For example, Wolff [17] developed a polarization imaging system composed of a polarization splitting prism and two CCD cameras to analyze the polarization state of specular reflection light on the object surface. In terms of enhancing contrast, Li [18] explored the potential of active polarization imaging technology in various underwater applications. He fully utilized the polarization characteristics of the target reflected light. Exponential functions were introduced to reconstruct cross-polarized backscatter images. The proposed method demonstrated an improvement for high-polarization objects under various turbidity conditions. Mo [19] proposed a method to calculate the polarization characteristics image that can reflect the differences in polarization characteristics of different materials. They fused the multi-angle orthogonal differential polarization characteristics (MODPC) image with the intensity image. The fused polarization image effectively enhanced the object detection information. This provided a basis for object classification, recognition, and segmentation. Umeyama [20] captured images of the measured object from different polarization angles by rotating the polarizer and separated the specular reflection components from the diffuse reflection components through independent component analysis. Overall, polarization imaging technology has certain advantages in reducing the impact of specular reflection light on the object surface, enhancing contrast, and improving imaging quality. Le Wang et al. [21] proposed a line-structured light imaging method for rail profiles based on polarization fusion. They proposed obtaining polarization component images and total intensity images of the rail laser section from multiple angles using a polarized camera. They solved the problem of local overexposure of laser images caused by specular reflection on the rail grinding surface through polarization fusion imaging technology. However, they did not discuss the existing problem of local underexposure.

Statistic features of multiple images are effectively extracted based on information complementarity after the image fusion. This compensate for the information insufficiency problems of single images, such as image information interference by noise, and too little image information. The result is more accurate information about the measured object. In consideration of the information correlation and complementarity between polarization images, researchers have proposed a variety of polarization image fusion methods, mainly including the frequency domain image fusion and spatial domain image fusion. In terms of frequency domain image fusion, Zhang Jingjing et al. proposed decomposing an image into low-frequency and high-frequency parts of different scales using the discrete wavelet transform (DWT). The wavelet coefficients of the fusion image are determined based on the wavelet coefficients of the low-frequency and high-frequency images as the statistical features [22]. Qiao Juan put forward a polarization image fusion algorithm based on the two-dimensional DWT, to enhance image details and improve the visual effect of images [23]. In terms of spatial domain image fusion, Yin Haining et al. proposed a polarization image fusion method based on feature analysis. Through this method, the fusion weight of an image can be calculated according to its gray feature, texture feature and shape feature. In addition, image fusion is used to solve the problem of detail loss that occurs when the polarization parameter image is calculated [24]. Recently, some image fusion methods based on deep learning, such as DPFN [25] and Gan [26], have become research hotspots. However, they are usually specific to natural scenes with rich color and texture features, while laser stripe data are relatively insufficient and lacks rich texture and color features. Therefore, these methods are not suitable for fusion of laser polarized stripe images.

To solve the above problems, based on previous research results, this paper proposes an improved rail profile measurement method based on polarization imaging. Specifically, a polarized camera is first used to capture the polarization component images of the rail laser section from multiple angles. Then, the polarization information of the rail laser section is extracted, and the Stokes parameter images, linear polarization angle images, and linear polarization degree images are calculated. Based on the S-RANSAC algorithm, the rail profile data corresponding to multiple polarization component images are fused. The fused data are used as the final rail profile measurement result. This method effectively improves the accuracy of rail profile measurement results under complex working conditions.

The structure of this paper is as follows: Section 2 introduces the traditional rail profile measurement methods and the exposure anomaly problems they face. Section 3 describes the rail profile measurement model based on polarization fusion. This includes the basic principle of polarization imaging, rail polarization component images, and the polarization data fusion method. Section 4 presents the experimental results, including laboratory static experiments and field dynamic experiments, and makes comparisons with other methods. Finally, Section 5 summarizes the conclusion of this paper.

## 2. The Structured Light Measurement Model and Exposure Anomaly Problem

### 2.1. The Structured Light Measuring Model

**Figure 1:**
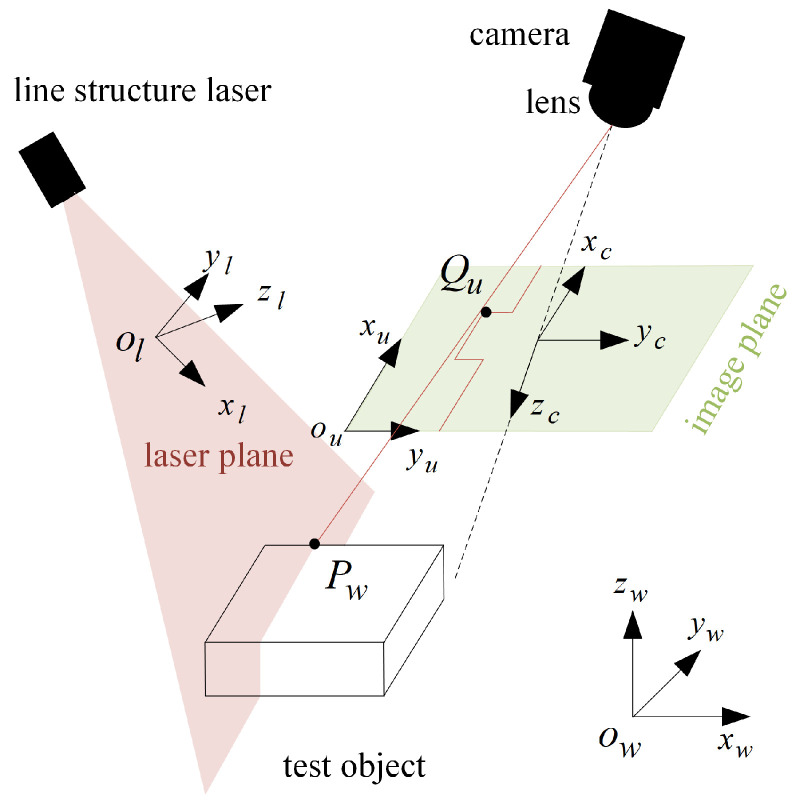
The geometric model of line-structured light perspective projection.

Figure 1 shows the perspective projection geometric model of the line-structured light profile measurement technology. The line-structured light profile measurement components are composed of the line laser, lens and camera. The line-structured light incident on the measured object surface is modulated into a light stripe reflecting the profile information of the measured object. The laser section image of the measured object is captured by photographing the light stripe. The actual profile of the measured object is calculated based on the pixel coordinates of image light stripe center, pixel coordinates of light stripe center, and system calibration parameters.

Before the rail profile measurement, the measurement system needs to be calibrated to obtain the internal parameters of cameras on both sides. It is also important to determine the parameters of laser planes on both sides. As shown in Figure 1, $o_{w}x_{w}y_{w}z_{w}$ is the world coordinate system, $o_{c}x_{c}y_{c}z_{c}$ represents the camera coordinate system, $o_{l}x_{l}y_{l}z_{l}$ denotes the laser plane coordinate system, and $o_{u}x_{u}y_{u}$ indicates the pixel coordinate system. $P_{w}={\left(x_{w},y_{w},z_{w},1\right)}^{T}$ refers to the coordinate (in the world coordinate system) of $P_{l}$ in the laser plane, and $Q_{u}={\left(x_{u},y_{u},1\right)}^{T}$ represents the image point corresponding to $P_{l}$. The expression based on the pinhole imaging model is as follows.

(1) $$sQ_{u}=A\left[R,t\right]P_{w}$$

where *s* is the scale factor, matrix A refers to the internal parameter matrix of the camera, and $\left[R,t\right]$ denotes the external parameter matrix of the camera, representing the rotation matrix and translation vector from the world coordinate system to the camera coordinate system respectively. In addition, $P_{w}$ in the laser plane meets the requirements of the following laser plane equation.

(2) $$ax_{w}+by_{w}+cz_{w}+d=0$$

where a,b,c,d represent the laser plane parameters. The mathematical model of line-structured light profile measurement can be obtained through combining Equations (1) and (2).

(3) $$\left\{\begin{array}{c} sQ_{u}=A\left[R,t\right]P_{w}\\ ax_{w}+by_{w}+cz_{w}+d=0 \end{array}\right.$$

Calculate the internal parameter matrix, external parameter matrix and laser plane parameters of the camera according to the method specified in the literature [27] first, then calculate $P_{w}$ according to the Equation (3), and finally obtain the actual profile of the measured rail.

### 2.2. Abnormal Exposure Issues

**Figure 2:**
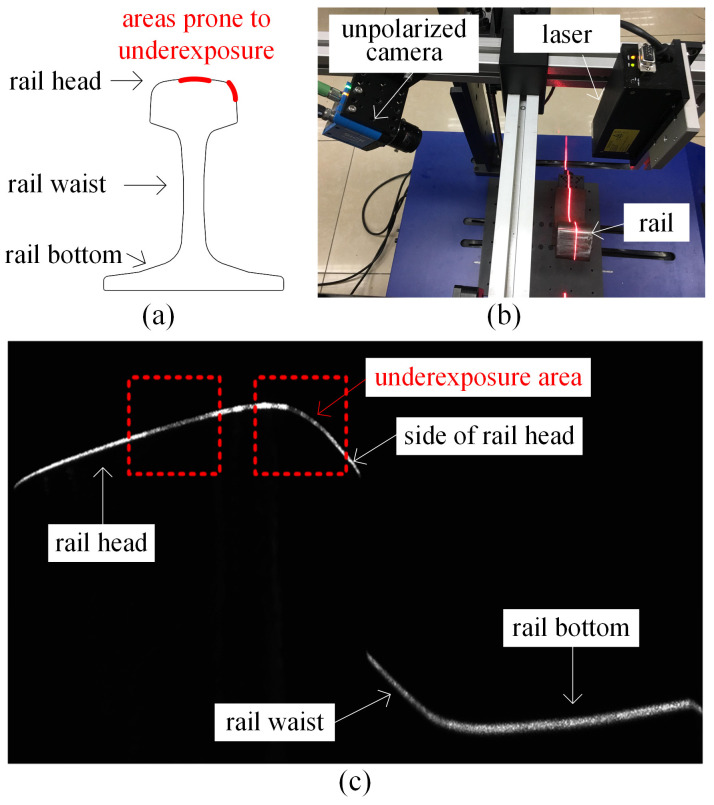
Local underexposure of the rail laser section image. (**a**) Rail areas prone to underexposure; (**b**) image acquisition device; (**c**) local underexposure image.

**Figure 3:**
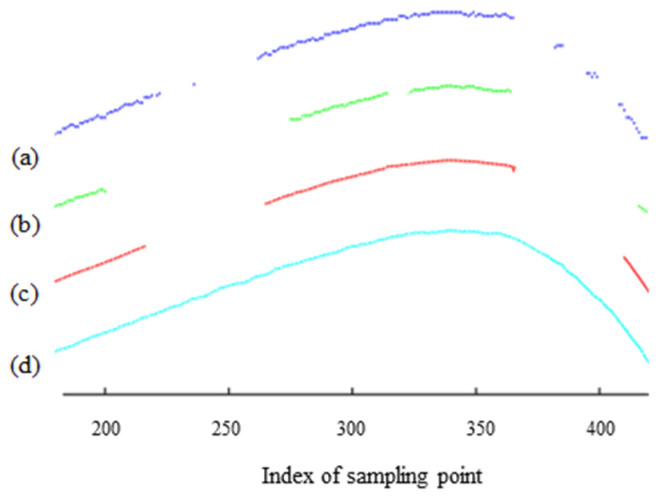
The center-of-light stripe in the underexposed area of the rail laser section image. (a) The maximum value algorithm; (b) the center-of-gravity algorithm; (c) the Steger algorithm; (d) the Miniprof profilometer.

Figure 2 shows the intensity image of the rail laser section captured by the traditional profile measurement method based on an unpolarized camera. The light stripe area of the rail head and the area near the rail gauge point are prone to low contrast and underexposure. This is because of the smooth surfaces and curvature changes in these areas, which result in a lack of diffuse reflection components in the camera. These factors lead to insufficient light reflection, making it difficult for the camera to capture clear images. This issue is highlighted in the dotted rectangular box in Figure 2c. Figure 3 shows the results of light stripe center extraction from the area in the dotted rectangular box in Figure 2. Here, (a), (b), and (c) represent the maximum value algorithm [28], the center-of-gravity algorithm [29], and the Steger algorithm [30] respectively, while (d) indicates the result of mapping the measurement data of the Miniprof profilometer to the image coordinate system. The Miniprof profilometer can achieve high-accuracy measurement because it directly contacts the test object. Its measurement results can be used to compare the effects of different light stripe center extraction algorithms. The comparison results show that in the normal intensity image area of the rail laser section, the rail profiles obtained by different light stripe center extraction algorithms are basically the same as those obtained by the Miniprof profilometer. In the underexposed area of the image, due to weak light stripe energy and low contrast, the light stripe center obtained by any light stripe center extraction algorithm is interrupted to varying degrees. The interrupted light stripe center cannot reflect the actual profile of the rail, resulting in the loss of rail profile data and affecting the rail profile registration. Severe underexposure will lead to the loss of data near the rail gauge point. Since the rail gauge point is a characteristic point for rail wear measurement, rail wear measurement may fail when this characteristic point is missing. The imaging quality and accuracy of traditional rail profile measurement based on intensity information need to be improved. Prolonging the exposure time can alleviate local underexposure. However, it causes overexposure of light stripes in the normal area. This undermines the accuracy of light stripe center positioning in the normal area. Therefore, the problem of local underexposure of rail laser section images cannot be effectively solved only by adjusting the exposure time.

## 3. The Measurement Principle

### 3.1. Polarization Imaging

Through polarization optical imaging, multiple intensity images of the measured object in different analyzer directions can be captured. Polarization information of the measured object can also be acquired. In other words, intensity information and polarization information of the measured object can be collected simultaneously. In contrast, traditional imaging technology is mainly used to capture intensity images of the measured object, without polarization information. For convenience, the cameras through which both intensity information and polarization information of the measured object can be acquired are collectively referred to as polarized cameras. Cameras through which only intensity information can be acquired are referred to as unpolarized cameras.

**Figure 4:**
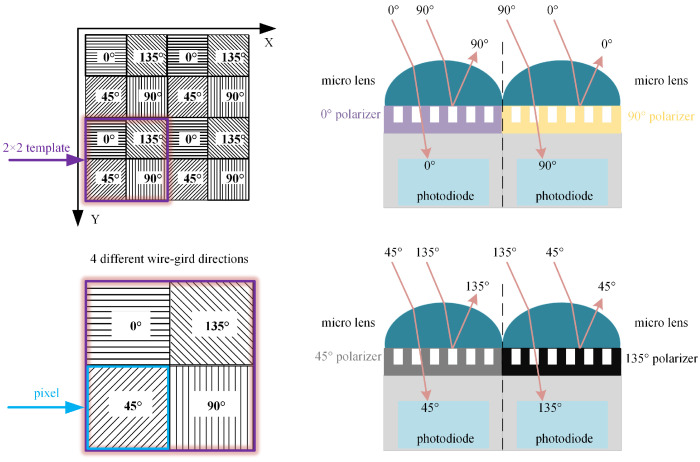
Polarization filters and pixel distribution of polarized camera.

A polarized camera is equipped with four polarization filters in different directions, which can simultaneously capture the polarization component images of the rail laser section from four directions. These images are abbreviated as four-directional polarization component images. Figure 4 shows the polarization filters and pixel distribution of the polarized camera. The four polarization filters of the polarized camera are arranged in a 2 × 2 configuration. Sub-pixels in the 2 × 2 template correspond to nanowire grating polarization filters at 0 degrees, 135 degrees, 45 degrees, and 90 degrees, respectively. The polarized light whose vibration direction is perpendicular to the nanowire grating will pass through the filter, while the polarized light whose vibration direction is parallel to the nanowire grating will be filtered out. All sub-pixels of the 2 × 2 template in the same polarization direction constitute a polarization component image. The gray values of all the sub-pixels at the same position in the 2 × 2 template are extracted to obtain four polarization component images that are 1/2 of original images in width and height. The four images are marked as $I_{0}$,$\;I_{45}$, $I_{90}$ and $I_{135}$ respectively. According to the Stokes representation method of polarized light, total intensity image $I_{t}$ is expressed below:

(4) $$I_{t}=I_{0}+I_{90}=I_{45}+I_{135}$$

In fact, the total intensity image is an intensity image captured through the traditional method and an ordinary intensity camera. Each pixel in a polarization component image is derived from the same 2 × 2 template. Polarization component images are pixel-aligned. Therefore, the total intensity image, generated by the superposition of polarization component images, is also pixel-aligned with the polarization component images.

According to reference [16], both the normal area and the overexposed area of the light stripe have partially polarized light with the same polarization angle. The interference part of the overexposed area has a high degree of polarization, while the non-interference part of the overexposed area and the normal area have a relatively low degree of polarization. Therefore, the four polarizers of the polarized camera in the orthogonal transmission direction can be used to filter out the interference components with a higher degree of polarization in the overexposed area. However, because the four-directional polarization component images have the effect of polarization filtering, this method cannot solve the problem of local underexposure of the rail laser section image. In this case, the polarization information of the rail laser section needs to be further extracted.

According to electromagnetic theory, light is defined as a transverse wave, and its electric field direction and magnetic field direction are perpendicular to the direction of propagation. In a plane perpendicular to the direction of light propagation, the electric vector may have different vibration states, which are also known as the polarization states of light. According to the polarization state, light can be further divided into natural light, partially polarized light, and fully polarized light. Fully polarized light is further divided into elliptically polarized light, linearly polarized light, and circularly polarized light. The Stokes vector S can be used to describe the polarization state of any light. The relationships between each component of the Stokes vector and the amplitude components $E_{x}$, $E_{y}$ of the light’s electric vector and the phase difference are shown in Equation (5).

(5) $$\left[\begin{array}{c} S_{0}\\ S_{1}\\ S_{2}\\ S_{3} \end{array}\right]=\left[\begin{array}{c} E_{x}^{2}+E_{y}^{2}\\ E_{x}^{2}-E_{y}^{2}\\ 2E_{x}E_{y}\cos\delta \\ 2E_{x}E_{y}\sin\delta \end{array}\right]$$

where $S_{0}$ represents the total intensity of light, $S_{1}$ denotes the light intensity difference between the linear polarization component of light wave in the x direction and the linear polarization component in the y direction, $S_{2}$ refers to the light intensity difference between the linear polarization component of light wave in the 45 degree direction and the linear polarization component in the 135 degree direction, and $S_{3}$ represents the light intensity difference between the left-handed circular polarization component and the right-handed circular polarization component. Natural light is usually partially polarized light, while partially polarized light can be regarded as a combination of fully polarized light and natural light. The first three components of the Stokes vector S can be expressed as follows:

(6) $$\left[\begin{array}{c} S_{0}\\ S_{1}\\ S_{2} \end{array}\right]=\left[\begin{array}{c} I_{0}+I_{90}\\ I_{0}-I_{90}\\ I_{45}-I_{135} \end{array}\right]$$

The linear polarization degree ***DoLP*** can represent the proportion of linearly polarized light in the partially polarized light. The linear polarization degree is represented in the Equation (7). The linear polarization angle ***AoP*** is the angle between the long axis of polarization ellipse and the x axis, namely the angle between the strongest vibration direction and the x axis. The expression of ***AoP*** is shown in the Equation (8).

(7) $$DoLP=\frac{\sqrt{S_{1}^{2}+S_{2}^{2}}}{S_{0}^{2}}$$

(8) $$AoP=\frac{1}{2}\tan^{-1}\frac{S_{2}}{S_{1}}$$

Polarization information of the measured object mainly involves the linear polarization components in various directions, Stokes vector, linear polarization degree ***DoLP***, and linear polarization angle ***AoP***. Through a traditional camera, only the intensity information of the measured object, namely the first component of the Stokes vector, can be acquired. In contrast, a polarized camera based on the polarization imaging technology makes it possible to obtain all the above polarization information. Therefore, rail profile information collected through a polarized camera is much more than that obtained through a traditional camera. Being capable to collect both polarization information and intensity information, a polarized camera is often used to enhance the contrast and reduce the impact of specular reflection light.

### 3.2. Polarization Component Images of Rail

**Figure 5:**
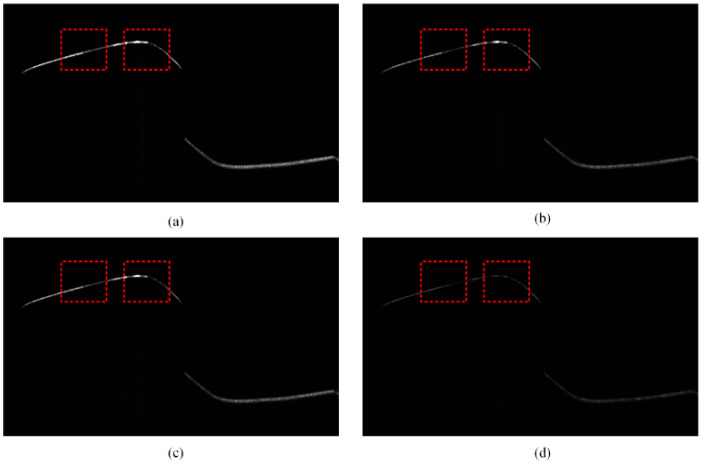
Four-directional polarization component image of the rail laser section. (**a**) 0 degree; (**b**) 135 degree; (**c**) 45 degree; (**d**) 90 degree.

**Figure 6:**
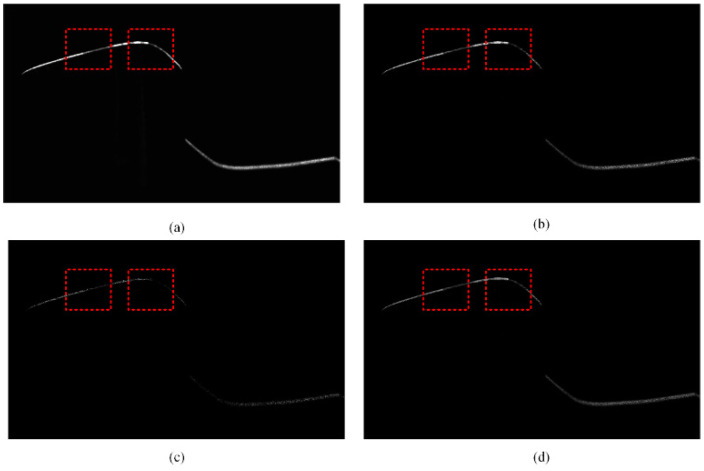
Stokes parameter image and linear polarization angle image. (**a**) $S_{0}$; (**b**) $S_{1}$; (**c**) $S_{2}$; (**d**) ***I_AoP_***.

**Figure 7:**
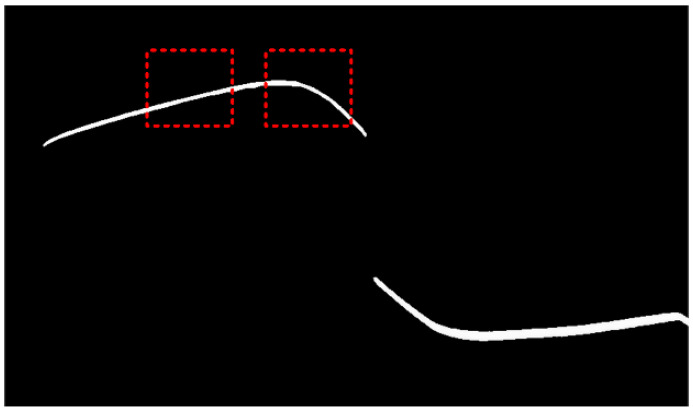
Linear polarization degree image.

The same position of the rail shown in Figure 2 was photographed with a polarized camera, to obtain four polarization component images of the rail laser section, as shown in Figure 5, the red box represents the same position as the red box in Figure 2. Then, the Stokes parameter images $S_{0}$, $S_{1}$ and $S_{2}$, linear polarization degree image ***I_DoLP_***, and linear polarization angle image ***I_AoP_*** were obtained based on the results of calculation according to the Equations (6), (7) and (8) respectively, as shown in Figure 6 and Figure 7. The red box represents the same position as the red box in Figure 2.

Polarization component images are pixel-aligned. Therefore, the synthesized Stokes parameter image, linear polarization degree image, and linear polarization angle image are also pixel-aligned. These images can reflect the rail profile information to a certain extent. There is a certain information correlation between them. Stokes parameter image $S_{0}$ is an intensity image captured through the traditional rail profile measurement method, and is equivalent to that in Figure 2c. The low gray value of the underexposed area of Stokes parameter image $S_{0}$ leads to the low confidence of the light stripe center. In contrast, the corresponding area of the linear polarization degree image is characterized by the strong light stripe energy. It also exhibits high imaging contrast and light stripe center confidence. This shows a certain degree of information complementarity.

The four-directional polarization component image, Stokes parameter image, linear polarization degree image, and linear polarization angle image are pixel-aligned with each other. They are correlated and complementary in rail profile information. Therefore, fusion of the aforesaid images to obtain the rail laser section images on the principle of reducing the fusion weight of the underexposed area and improving the fusion weight of the normal area. This approach can better solve the problem of underexposure of light stripe images captured through the traditional rail profile detection technology.

**Figure 8:**
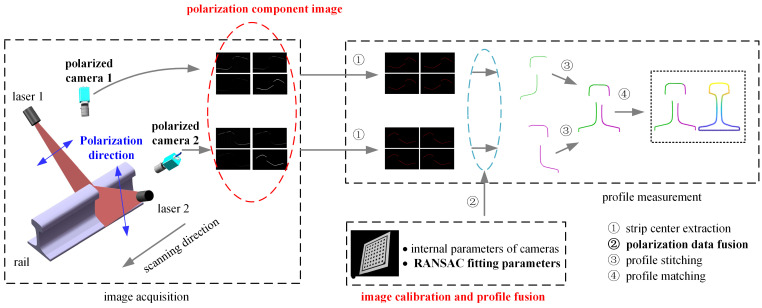
Schematic diagram of the rail profile measurement based on polarization imaging.

Based on the above analysis results, this paper proposes a rail profile measurement method based on polarization imaging, as shown in Figure 8. Specifically, the laser is equipped with a linear polarizer to obtain the linearly polarized light in the laser plane in the vibration direction, and the polarization direction of the laser is shown in the figure. In addition, a polarized camera is used to capture the polarization component images of the rail laser section from multiple polarization angles. Stokes parameter images $S_{0}$, $S_{1}$ and $S_{2}$, linear polarization degree image ***I_DoLP_***, and linear polarization angle image ***I_AoP_*** are synthesized from such polarization component images. The aforesaid images are fused through the image fusion algorithm to obtain rail laser section images, which lays a foundation for light stripe center extraction, calibration, profile stitching, profile registration, and final rail profile measurement.

### 3.3. The Polarization Data Fusion Method

The determination of fusion weights is crucial for improving the quality of multi- polarized light image fusion. Wang [21] introduced a light stripe reliability evaluation mechanism to determine the fusion weights of source images. For light stripe reliability evaluation, statistical features such as light stripe width, gray value, and average residual sum of squares were used as evaluation indicators. For each source image, the light stripe reliability in each column needed to be calculated. The total pixel intensity or light stripe width was separately selected as an evaluation index to calculate the reliability of stripe polarization imaging, and the weights of each component image were continuously adjusted to achieve the optimal fusion effect. Additionally, these two evaluation indexes could also be used comprehensively to calculate the light stripe reliability and obtain image fusion weights. This method effectively overcomes the problem of rail surface reflection. However, when applied to the dynamic measurement of the profile system, there are still some issues to be resolved:

(1) The fusion strategy and weight calculation lack systematic quantitative analysis, overly depending on qualitative analysis results and artificial experience thresholds.

(2) Light stripes change during train operation, and unpredictable changes may occur due to factors such as rail wear, sunlight, and foreign matter interference. Thus, using the light stripe width and brightness as criteria for determining fusion weights makes it difficult to adapt to the complex and ever-changing conditions of an entire railway line.

(3) The fusion calculation of multiple polarization images is time-consuming, thus affecting the real-time performance of the measurement system.

**Figure 9:**
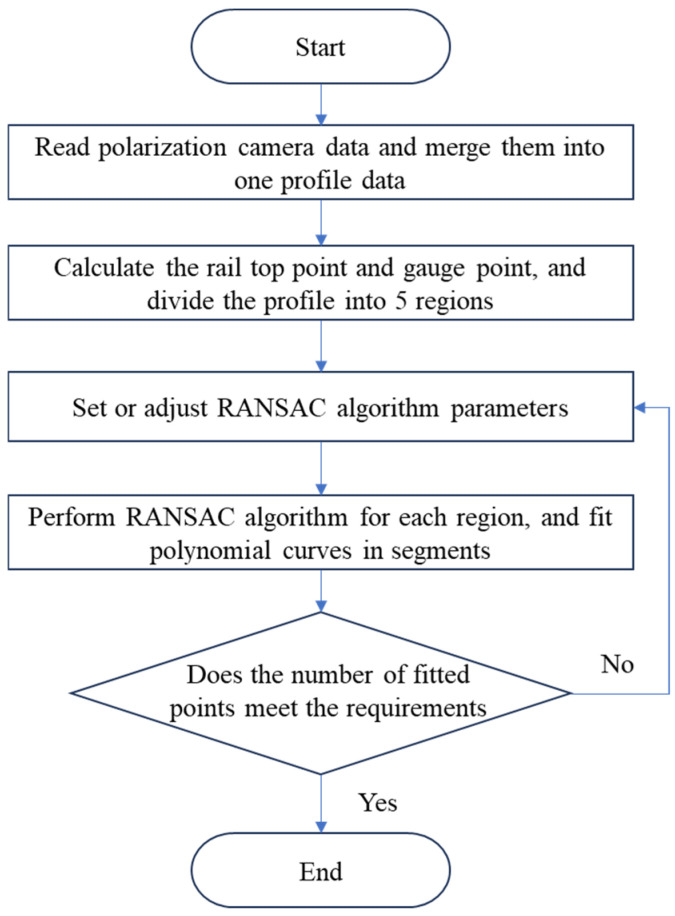
Flowchart of the polarization profile data fusion algorithm based on S-RANSAC.

To solve the above problems, and a data fusion algorithm for segmented RANSAC polarization point cloud is proposed in this paper, as shown in Figure 9.

The algorithm process is described as follows:

(1) For nine polarization component images represented by $I_{0}$, $I_{45}$, $I_{90}$, $I_{135}$, $I_{AoP}$, $I_{DoP}$, $S_{0}$, $S_{1}$ and $S_{2}$, solve the light stripe centers to obtain the corresponding polarization profile data, denoted as $P_{0}$, $P_{45}$, $P_{90}$, $P_{135}$, $P_{AoP}$, $P_{DoP}$, $S_{0}^{\prime }$, $S_{1}^{\prime }$ and $S_{2}^{\prime }$;

(2) According to point coordinates, merge nine pieces of polarization profile data into one piece of profile data denoted as

(9) $$P=\cup (P_{0},P_{45},P_{90},P_{135},P_{AoP},P_{DoP},S_{0}^{\prime },S_{1}^{\prime },S_{2}^{\prime })\;$$

where ∪(·) represents the fusion of profile coordinate data.

(3) Based on the top and gauge points, divide these profiles into five areas denoted as

(10) $$\left(P_{t},P_{S_{1}},P_{S_{2}},P_{w_{1}},P_{w_{2}}\right)=segment(P,T_{x,y},G_{x,y})$$

where segment(·) is a region-based segmentation, $T_{x,y}$ is the coordinate of the rail top, and $G_{x,y}$ is the coordinate of the rail gauge point. The division of the five regions incorporates strong prior information about rail shapes. By utilizing the rail top and gauge points, as well as the rail model, the rails can be precisely divided into the rail top region, rail head transition region, rail head side region, rail web region, and rail bottom region according to Formula (10). Each region possesses a relatively fixed curvature, ensuring consistent reflection of light.

(4) Implement the random sample consensus (RANSAC) for each region to obtain the best-fit polynomial equation, denoted as

(11) $$\left(C_{t},C_{s_{1}},C_{s_{2}},C_{w_{1}},C_{w_{2}}\right)=\left\{\begin{array}{l} RANSAC(P_{t},\xi_{t},m_{t},n_{t})\\ RANSAC(P_{s_{1}},\xi_{s_{1}},m_{s_{1}},n_{s_{1}})\\ RANSAC(P_{S_{2}},\xi_{S_{2}},m_{S_{2}},n_{S_{2}})\\ RANSAC(P_{w_{1}},\xi_{w_{1}},m_{w_{1}},n_{w_{1}})\\ RANSAC(P_{w_{2}},\xi_{w_{2}},m_{w_{2}},n_{w_{2}}) \end{array}\right.$$

where $C_{t},C_{S_{1}},C_{S_{2}},C_{w_{1}},C_{w_{2}}$ represent the polynomial curves of RANSAC fitting for five segments; $\xi_{t},\xi_{S_{1}},\xi_{S_{2}},\xi_{w_{1}},\xi_{w_{2}}$ are interior point thresholds, the samples with values less than such thresholds are used for fitting, while other samples with values greater than such thresholds are removed as noise; $m_{t},m_{S_{1}},m_{S_{2}},m_{w_{1}},m_{w_{2}}$ represent the times of sampling iterations; $n_{t},n_{S_{1}},n_{S_{2}},n_{w_{1}},n_{w_{2}}$ represent the optimal polynomial fitting powers for five segments, which can be obtained by using the least square method on the basis of selecting multiple pieces of typical profile data from actual railway line, and constructing a global optimization model.

(5) Stitch the fitting curves $C_{t},C_{S_{1}},C_{S_{2}},C_{w_{1}},C_{w_{2}}$ of the five segments into a complete half section profile of steel rail denoted as $C=stitching\left(C_{t},C_{S_{1}},C_{S_{2}},C_{w_{1}},C_{w_{2}\;}\right)$, where C is the optimal profile fitting curve formed after fusion of multiple polarization point cloud data.

## 4. Experimental Results

### 4.1. Laboratory Static Experiments

**Figure 10:**
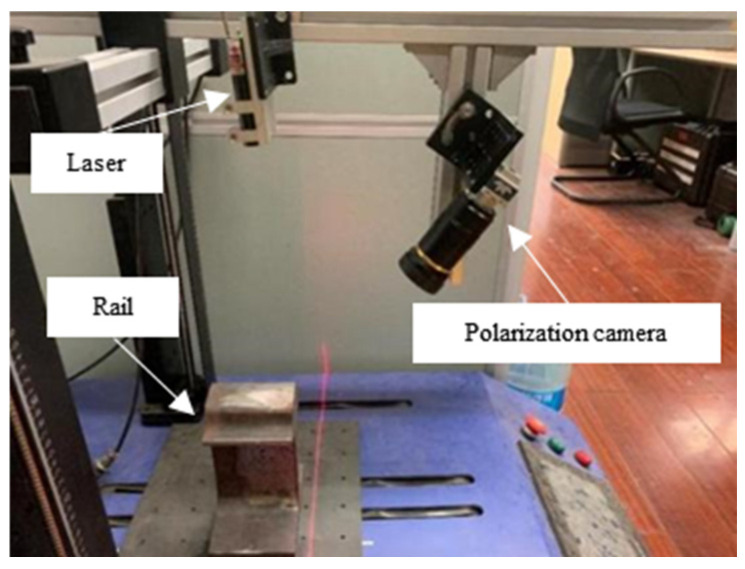
Experimental setup.

The rail profile measurement device was constructed, as shown in Figure 10. The rail shown in Figure 2 was placed on the electronic control translation platform. The Genie Nano M2450 polarized camera manufactured by Teledyne Dalsa, a Canadian company headquartered in Waterloo, Ontario, was selected to photograph the same position of the rail in Figure 2. The laser cross-sectional images of the rail were acquired at equal intervals of 2 mm. The main parameters are shown in Table 1.

**Figure 11:**
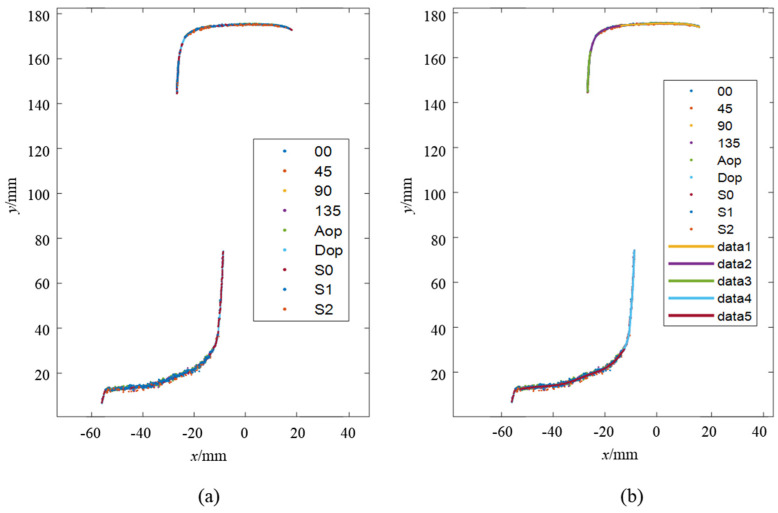
Experimental result of S-RANSAC: (**a**) fused point cloud points; (**b**) segmented polyfit result.

This process enabled the acquisition of the four-directional polarization component images of the rail laser section. Then, Stokes parameter images $S_{0}$, $S_{1}$ and $S_{2}$, linear polarization degree image ***I_DoLP_***, and linear polarization angle image ***I_AoP_*** are calculated and obtained respectively according to the Equations (6), (7) and (8), as shown in Figure 6 and Figure 7. We extracted the light stripe center using the gray centroid method [29]. The proposed S-RANSAC algorithm was then employed to fuse each polarization component, and the outcomes are illustrated in Figure 11. Data 1 to data 5 represent the partition fitting results, which are respectively the rail top region, rail head transition region, rail head side region, rail web region, and rail bottom region. By comparing with Figure 3, it becomes evident that the traditional method incurs data loss in the rail head area. In contrast, the proposed method is capable of obtaining effective contour data in the rail head area.

**Figure 12:**
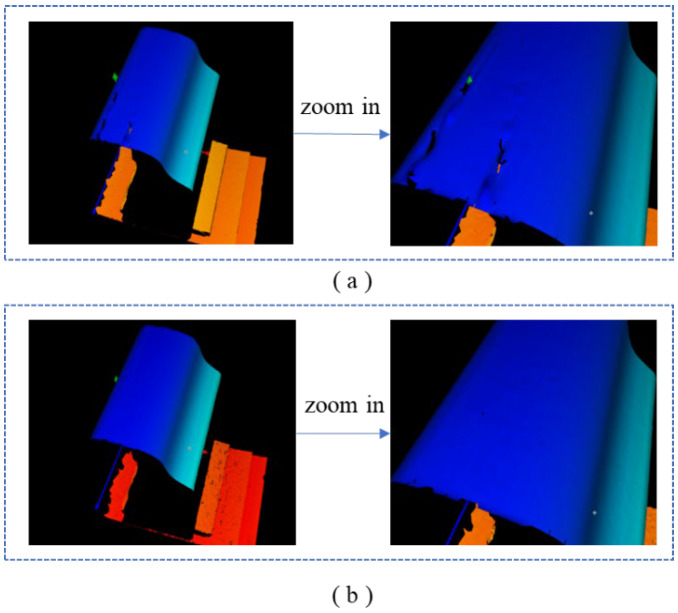
3D reconstruction of rail: (**a**) the traditional method; (**b**) the proposed method.

To further verify the effectiveness of the proposed method for the three-dimensional reconstruction of rails, the proposed method was utilized to perform 3D reconstruction of the measured rail. Meanwhile, the profile of the rails obtained from the Stokes parameter image $S_{0}$ was regarded as the measurement result of the traditional method. Figure 12 presents the 3D reconstruction results of the steel rails using the traditional method and the proposed method respectively. It can be clearly observed that the traditional method led to partial data loss in the 3D-reconstructed steel rails due to abnormal exposure. Conversely, the proposed method did not exhibit any data loss in the 3D-reconstructed steel rails, and the reconstruction results still accurately reflected the true condition of the steel rails.

### 4.2. The On-Site Dynamic Test

**Figure 13:**
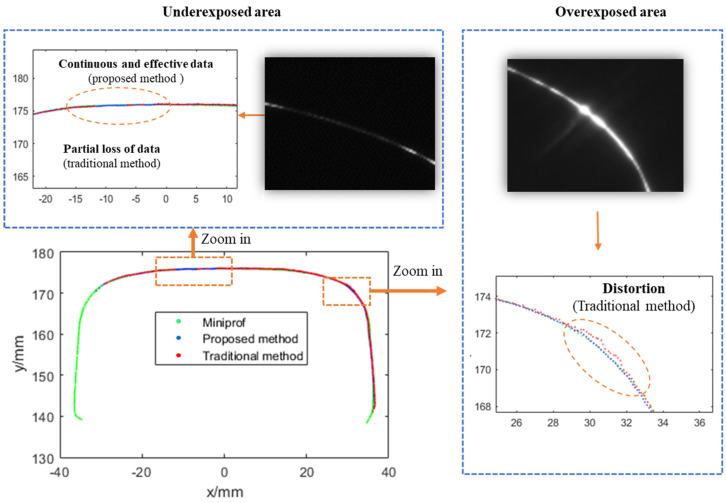
Comparison of rail profile measurement results between the proposed and traditional methods.

The on-site dynamic test was carried out near Qinghecheng Station at K 346 on the Beijing–Kowloon Line. The Beijing–Kowloon Line is a trunk railway with shared passenger and freight traffic. Due to its high traffic volume and diverse cargo types, the on-site operational conditions are relatively complex: the rail surface is often relatively shiny and mixed with foreign objects, which affects image quality. The profiles of the tested railway line include situations such as web burial of the rail, ambient light interference, and the polished rails. Miniprof, a contact-type rail profile measurement device with a measurement accuracy of 0.02 mm, was used as a reference. The rail profile obtained from the Stokes parameter image was considered as the measurement result of the traditional method. Given the difference in the number of points collected by the measurement system and the number of points obtained by Miniprof, it was necessary to perform smoothing processing before discretizing the profile. This ensured that corresponding point pairs could be compared, thereby enabling a genuine evaluation of the profile difference. Figure 13 shows a comparison diagram of the rail profile at one typical position. The figure contains two enlarged areas, namely the underexposed area at the top of the rail (in the upper left corner of the figure) and the overexposed area on the side of the rail head (on the right side of the figure). In the underexposed area, partial data loss occurred (the red dots are interrupted) when using the traditional method. However, since the proposed method acquires more polarization information through the polarization imaging technology and adopts a fusion method based on divided regions, continuous and valid contour data can still be obtained in this area, and there is a high degree of consistency with the contour data obtained by Miniprof. Similarly, in the overexposed area, data distortion and deformation occurred (the red dots deviate significantly from the green dots) with the traditional method. Nevertheless, the contour data obtained by the proposed method still show a high degree of consistency with that obtained by Miniprof. Thus, it can be demonstrated that the proposed method can simultaneously address the issues of both underexposure and overexposure.

**Figure 14:**
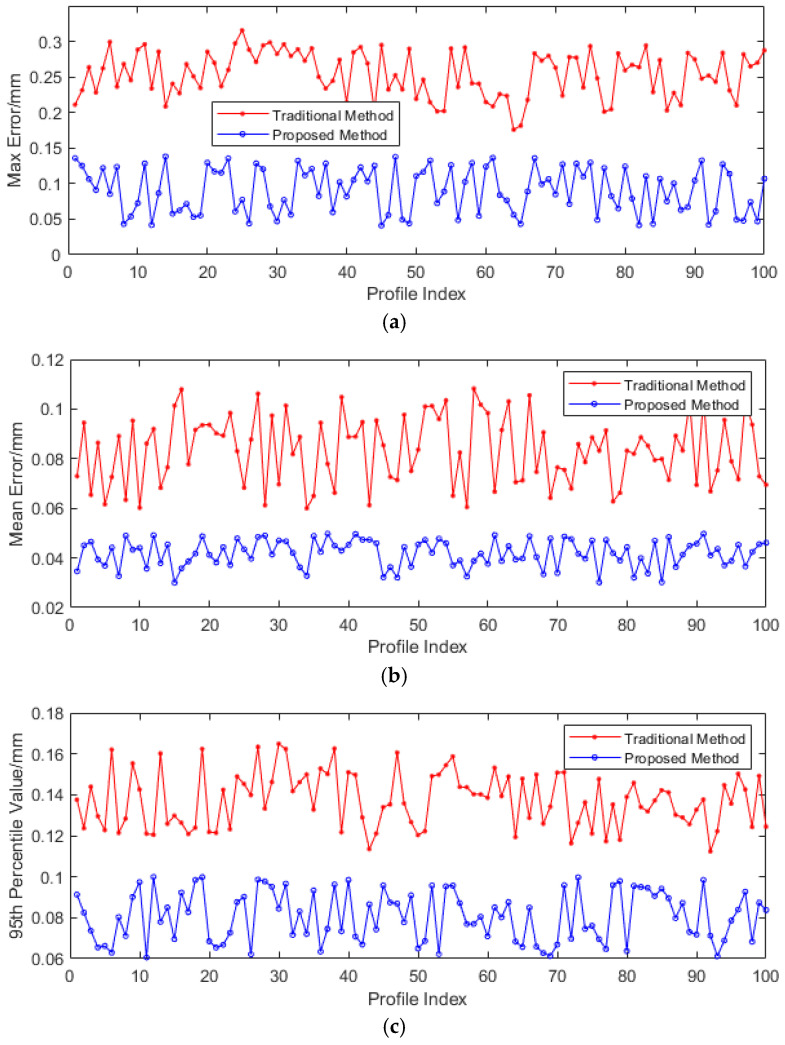
The measurement error of rail profile between the proposed and traditional methods. (**a**) **ME**, (**b**) **AE**, and (**c**) **PE**.

To quantitatively compare the accuracy of the rail profile measurement of the proposed method, 100 sets of rail profile data were collected using Miniprof. For each sampling point, the Miniprof measurement data were taken as the true value, and the differences between the rail profile data obtained by the traditional method and the proposed method and the Miniprof data were statistically analyzed. The statistical parameters included the maximum error (**ME**), average error (**AE**), and 95th percentile error (**PE**). The results are presented in Figure 14, and Table 2 shows the average values of the three statistical measures mentioned above. Compared with the traditional method, the maximum, average, and 95th percentile values of the rail profile measurement errors have all decreased to varying degrees. Taking the 95th percentile value of the rail profile measurement error as an example, it has significantly declined from 0.137 mm, as recorded in conventional methodologies, to 0.081 mm. This represents a remarkable reduction of 40.9%. This clearly demonstrates the enhanced precision of the rail profile data obtained through the proposed method, which now more accurately represents the true profile of the rail.

**Figure 15:**
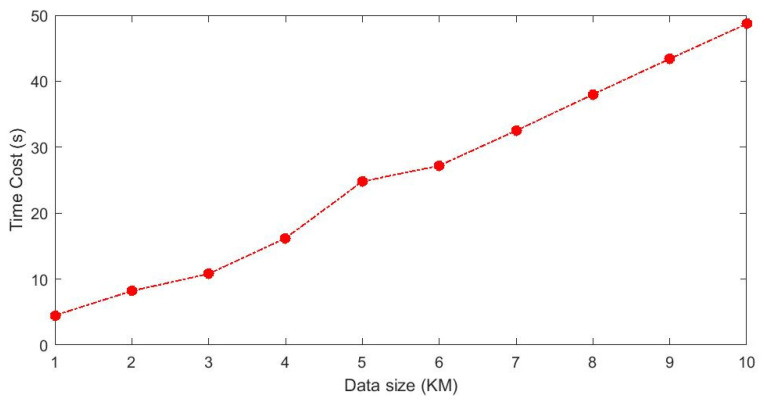
The relationship between algorithm execution efficiency and data size.

To verify the execution efficiency of the S-RANSAC algorithm, typical profile data were selected for testing. The computer CPU parameters were Intel(R) Core(TM) i7-10700K CPU @ 3.80 GHz, and the programming language used was C++ (version 20). To improve the efficiency of algorithm execution, a programming architecture for data concurrency was designed. The 4000 sets of profile data collected per kilometer were divided into 10 concurrent queues, with each queue buffering 400 sets of profile data. The relationship between algorithm execution efficiency and data size is shown in Figure 15. Due to the randomness of RANSAC, the time consumption may vary slightly each time, but it can process approximately 200 m of data per second.

### 4.3. Comparison with Other Research Methods

**Figure 16:**
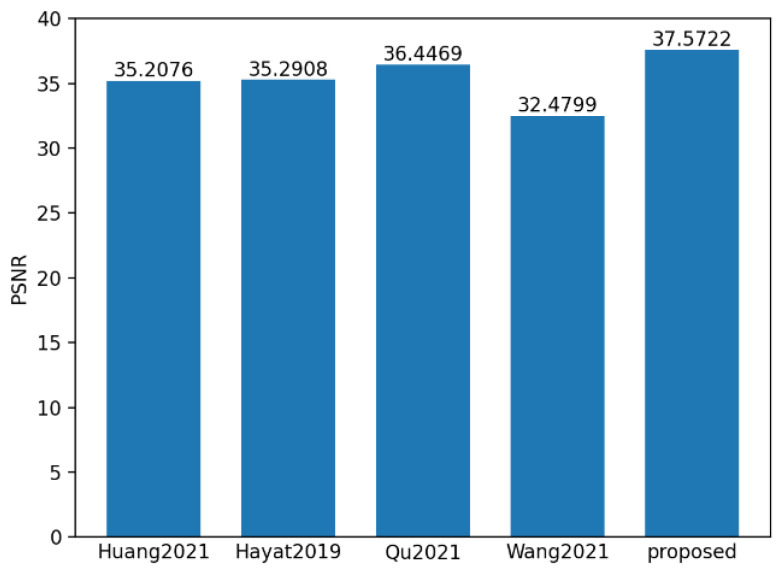
Mean value histogram of **PSNR** indicators obtained by the five fusion methods.

**Figure 17:**
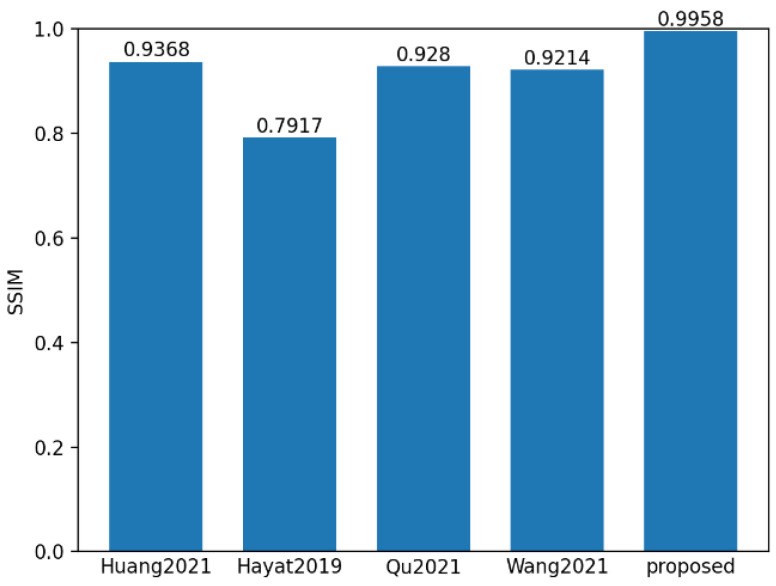
Mean value histogram of **SSIM** indicators obtained by the five fusion methods.

**Figure 18:**
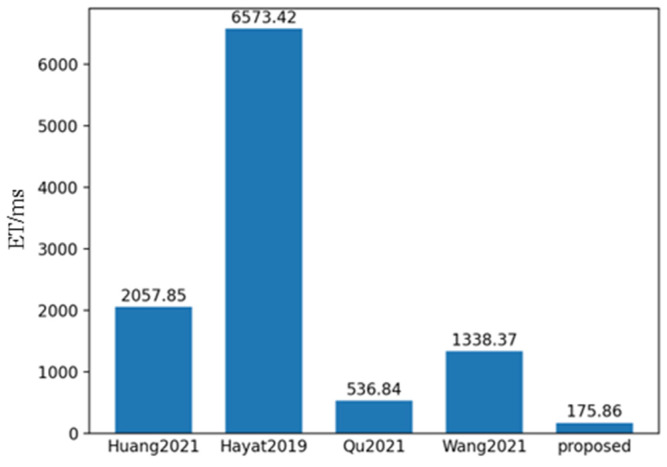
Mean value histogram of algorithm time obtained by the five fusion methods.

To evaluate the performance of the proposed method, 550 sets of polarization component images of steel rails were randomly selected, and the proposed method, along with the methods in references Huang [31], Hayat [32], Qu [33], and Wang [34], were used for fusion analysis. **SSIM** and **PSNR** are the most commonly used indicators for evaluating multi-exposure fusion algorithms in dynamic scenes. The **PSNR** indicator measures the similarity between the fused image and the source images in terms of image gray levels. A larger **PSNR** means that the fused image is close to the source images and has less distortion. Therefore, the larger the **PSNR** value, the better the fusion performance. **SSIM** is used to model image losses and distortions, to which the human visual system is sensitive. It consists of three parts, namely correlation, luminance, and contrast distortion. The **SSIM** between the source image and the fused image is defined as the product of these three parts. **SSIM** reflects the degree to which the fused image preserves the local structural details of the input images. The larger the value of this indicator, the greater the degree of structural preservation, and the better the fusion effect. **ET** represents the algorithm execution time. As shown in Figure 16, Figure 17 and Figure 18, it can be observed that the proposed method achieved the highest scores in both **PSNR** and **SSIM**, which were 37.57 and 0.99 respectively. Compared with the **PSNR** of 36.44 and the **SSIM** of 0.928 of the method proposed by Qu, who ranked second, and the execution time of 536.84 ms, the proposed method is not only better in maintaining data quality and structural integrity, but also more efficient in algorithm execution, with the time being only 175.86 ms, reflecting its efficient processing ability and good fusion effect.

On this basis, we compared the proposed S-RANSAC method with the above-mentioned mainstream fusion methods before and after the sub-area division. The experimental results are shown in Table 3. In line with Table 3, S-RANSAC has a more competitive performance under **PSNR**, **SSIM** and time efficiency. In consequence, it is selected as the for polarized component fusion task.

## 5. Conclusions

A novel rail profile measurement method founded on multi-polarization fusion has been presented to resolve the issue of insufficient local exposure in laser cross-section images, which is a common hurdle in traditional rail profile measurement techniques. This advance involves the creation of a profile data fusion algorithm that utilizes the S-RANSAC algorithm, specifically designed for four-directional polarization component images, Stokes parameter images, linear polarization angle images, and linear polarization degree images. This approach effectively alleviates the problem of local underexposure in the laser cross-section of steel rails, securing a comprehensive and accurate depiction of the rail profile. Following three-dimensional reconstruction, the method guarantees that the steel rail no longer suffers from data loss, which is a crucial improvement over traditional methods. This innovation surmounts the exposure insufficiency in key areas of laser cross-section images of steel rails, which can significantly influence the extraction of light strip centers. By preserving the integrity of profile data in critical areas, the method boosts the accuracy and stability of profile detection under complex working conditions. This not only ensures the effectiveness of profile analysis, comparison, and evaluation but also facilitates the expansion of rail profile detection application scenarios.

Future research will probe into alternative image fusion methods, such as frequency-domain fusion or deep-learning-based fusion, with the objective of further enhancing algorithm efficiency and robustness. This could potentially result in more accurate and reliable rail profile measurements, even in the most demanding operating environments. Additionally, efforts will be made to optimize the existing S-RANSAC algorithm to reduce its computational complexity and improve its real-time performance, making it more suitable for practical applications in railway infrastructure inspection. Through these continuous improvements, the proposed method is expected to play an increasingly significant role in ensuring the safety and reliability of railway transportation systems.

## Figures and Tables

**Table 1:** The main parameters in laboratory static experiments.

Index	Item	Parameter
1	Camera	Resolution: 2464 × 056, Pixel Size: 3.45 µm × 3.45 µm
2	Lens	Focus: 12 mm
3	Laser	Power: 500 mw

**Table 2:** Statistical results of 100 sampling points (mm).

Measurement Error	Max Value	Average Value	95th Percentile Value
Traditional method	0.254	0.084	0.137
Proposed method	0.092	0.042	0.081

**Table 3:** Comparisons on polarization fusion algorithms.

Algorithm	PSNR (Single/Multi-Region)	SSIM (Single/Multi-Region)	Time (Single/Multi-Region)
Huang	34.21/35.15	0.94/0.98	2057.85 ms/3125.14 ms
Hayat	34.29/34.98	0.79/0.91	6573.42 ms/7402.27 ms
Qu	36.45/37.05	0.93/0.97	536.84 ms/1026.33 ms
Wang	32.48/33.93	0.9210.97	1338.37 ms/1579.43 ms
S-RANSAC	36.57/37.91	0.98/0.99	175.86 ms/341.10 ms

## Data Availability

Data underlying the results presented in this paper are not publicly available at this time but may be obtained from the authors upon reasonable request.
